# Optimal Dietary Fiber Intake to Retain a Greater Ovarian Follicle Reserve for Gilts

**DOI:** 10.3390/ani9110881

**Published:** 2019-10-29

**Authors:** Meng Cao, Yong Zhuo, Lechan Gong, Lianchao Tang, Zipeng Li, Yang Li, Min Yang, Shengyu Xu, Jian Li, Lianqiang Che, Yan Lin, Bin Feng, Zhengfeng Fang, De Wu

**Affiliations:** Key Laboratory for Animal Disease-Resistant Nutrition of the Ministry of Education of China, Animal Nutrition Institute, Sichuan Agricultural University, Chengdu 611130, China; caomeng1111@126.com (M.C.); Gongyuechanpenny@163.com (L.G.); steve5678t@163.com (L.T.); 15554422287@163.com (Z.L.); liyang_cc@yeah.net (Y.L.); yangmin040101@yeah.net (M.Y.); shengyu_x@hotmail.com (S.X.); lijian522@hotmail.com (J.L.); clianqiang@hotmail.com (L.C.); linyan936@163.com (Y.L.); fengbin@sicau.edu.cn (B.F.); fangzhengfeng@hotmail.com (Z.F.)

**Keywords:** dietary fiber, gilts, follicle atresia, ovarian follicle reserve, primordial follicle

## Abstract

**Simple Summary:**

Successful development of replacement gilts plays a critical role for sustainable swine production, whereas most gilts entering the breeding herd are culled at a young age. Recent advances in reproductive physiology revealed that the ovarian reserve could be considered as an important target of improving reproductive span. Therefore, in the present study, we hypothesized that ovarian follicle development and survival could be enhanced by dietary fiber. Currently there is no appropriate level of dietary fiber intake for growing replacement gilts despite the fact that dietary fiber, rather than starch, protein, or feeding allowance level, could improve the fertility of gilts. Results in the present study demonstrated that ovarian follicle development and survival were sensitive to dietary fiber levels. Our results shed light on the mechanisms underlying the beneficial effects of dietary fiber on the reproduction of sows and provided nutritional insights for enhancing the lifetime fertility of pigs by targeting the ovarian reserve.

**Abstract:**

Ovarian follicle activation and survival were recently found to be controlled by nutrient sensors AMP-activated protein kinase (AMPK) and mammalian target of rapamycin (mTOR) and apoptosis related markers Caspase-3, Bax, and Bcl-2, yet their expression as regulated by dietary fiber remained uncertain for gilts. To investigate the effects of dietary fiber levels on ovarian follicle development, and the cellular molecular components related to follicle activation and survival of gilts, 76 gilts with similar bodyweight and age were fed four diets, including a corn-soybean meal based control diet, or other three diets to consume 50%, 75%, and 100% more dietary fiber than the control gilts at different experimental phases. Inulin and cellulose (1:4) were added to the corn-soybean meal basal diet to increase dietary fiber content. The growth traits, and the age, bodyweight, and backfat thickness at puberty were not affected by diets. The number of primordial follicles and total follicles per cubic centimeter of ovarian tissue linearly increased with dietary fiber level at day 30 of the experiment and at the 19th day of the 3rd estrous cycle, without negatively affecting the formation of antral follicle with diameter between 1–3 mm or larger than 3 mm. These changes were associated with altered phosphorylation of mTOR, S6, Extracellular regulated protein kinases 1/2 (ERK1/2) and AMPK, and mRNA expression of Caspase-3, Bax, and Bcl-2 in ovarian tissues. Collectively, this study demonstrated a beneficial effect of dietary fiber on the ovarian follicle reserve in gilts, which provides a basis for enhancing reproduction in the short- or long-term.

## 1. Introduction

A large proportion of gilts are maintained in swine production for the renewal of the breeding herd, therefore emphasizing the importance of successful gilt development for overall herd performance and profitability. However, 40–50% of the replacement gilts entering the breeding herd suffer reproductive failure at a young age, and therefore provide only 30–40 piglets in their lifetime [1,2]. The culling of sows could be ascribed to many reasons but the reproductive disorders, such as estrous initiation failure, conception failure, and small litter size, account for most of the reasons for culling [1,2].

It is generally accepted that ovarian follicle reserve and quality of follicles are key determinants of the reproductive span across different mammal species, and it is quite clear that reproduction terminates as the ovarian follicle diminishes [3,4,5]. The ovarian reserve has been observed to be controlled by several factors [5], yet the environment factors (e.g., nutrition) controlling the ovarian reserve of female pigs remains largely unknown. Activation of the ovarian primordial follicle, the first step of folliculogenesis, is closely associated with cellular nutrient sensors AMPK or mTOR, and the survival of follicles is influenced by apoptosis related proteins including Bax, Bcl-2, and Caspase-3 [5]. Recently, research conducted in our lab discovered that ovarian follicle activation and survival in pigs and mice are sensitive to the dietary macronutrient balance, which resulted in differences of reproductive span [6]. There is growing attention to the application of dietary fiber to improve health in humans and animals, and results have found that increasing dietary fiber intake can reduce the risks of certain diseases such as gut disorders, diabetes, coronary heart disease, and cancer [7,8,9]. Previous research revealed that additional dietary fiber, but not feeding allowance level, protein, or starch supplements, was able to enhance the follicle quality and early embryonic survival in gilts [10]. Despite the beneficial effects of dietary fiber on the reproductive performance of replacement gilts [10,11,12], the optimal dietary fiber intake for replacement gilts remains uncertain. Currently, only a limited number of studies have focused on the effects of dietary fiber intake on reproductive performance in the replacement gilts, and the inclusion of dietary fiber was achieved by formulating high levels of fiber-rich ingredients, e.g., sugar beet pulp and lupin [10,11,13]. However, there are many different nutrients contained in those ingredients, and it is hard to assess if their beneficial effects were exclusively attributed to dietary fiber. Therefore, in the present study, the effects of dietary fiber levels on the follicular development for replacement gilts will be investigated using purified dietary fiber.

## 2. Materials and Methods

The present experiment was conducted at the Research Farm of the Animal Nutrition Institute, Sichuan Agricultural University, Ya’an, China. All animal procedures in this study were handled in accordance with Institutional Animal Care and Research Committee of Sichuan Agricultural University, and the ethical approved project identification code: SICAU-2015-034.

### 2.1. Animals and Diets

A total of 76 Landrace × Yorkshire crossbred gilts with similar body weights (33.8 ± 3.9 kg; Mean ± SD) and age (92.6 ± 0.6 days; Mean ± SD) were randomly allocated to one of four dietary groups (*n* = 19), including a control group (1.0 fold dietary fiber, 1.0 DF) which consumed a daily intake of 200.32, 262.92, 310.50, and 347.76 g dietary fiber during 1 to 30 days, 31 to 60 days, 61 to 120 days and 121 days to the end of the experiment, respectively. The other three groups consumed 50% (1.5 DF), 75% (1.75 DF), and 100% (2.0 DF) more dietary fiber than the gilts in the 1.0 DF group at different experimental phases. The basal diet was corn-soybean meal based and was divided into two phases including experimental day 1 to 60 and day 61 to the end, which contained 12.52% and 12.42% total dietary fiber, respectively (Table 1). The 1.0 DF gilts were fed 1.6, 2.1, 2.5, and 2.8 kg of the basal diet per day for 1 to 30 days, 31 to 60 days, 61 to 120 days and 121 days to the end of the experiment, respectively (Table 2). The detailed diet formulations and daily nutrient intake are presented in Table 1 and Table 2, respectively. In the present study, the definition of dietary fiber is different from that of crude fiber or neutral detergent fiber. The concentration of dietary fiber, sum of soluble and insoluble fiber, was measured by enzymatic-gravimetric method AOAC 991.43 with minor modification. The dietary fiber used in this trial is purified inulin (99%, Orafti GR, Tienen, Belgium), with an average degree of polymerization between 10 and 12, and cellulose (99%) from Guangxi Shangda Tech Co., LTD (Nanning, China). Inulin is water soluble and easy to be fermented in the proximal gut by the microbiota. Cellulose is insoluble to water and is not easy to be fermented by the distal gut microbiota. Inulin and cellulose were added at the ratio of 1:4, which was based on previous research [12]. Gilts were housed individually (2.0 m × 0.8 m) in a breeding facility and were fed twice daily at 08:00 and 14:30. Water was provided ad libitum. The environment temperature was controlled at 20 °C to 24 °C.

### 2.2. Measurement of Growth Traits and Pubertal Onset

Onset of estrous was detected by only one experienced stockperson based on behavioral and vulvar characteristics of gilts as previously described [12,14]. The first day of standing heat was considered pubertal estrous and defined as the first day of the estrous cycle. The body weight of gilts was measured at day 30, 60, 90, 120 of the experiment, at puberty, and at the 19th day of the 3rd estrous cycle. The backfat thickness was measured along with the measurement of body weight, which was detected at 65 mm on both sides of the dorsal mid-line at the last rib using an ultrasound scanner (Renco LeanMeater, Minneapolis, MN, USA). Both side measurements were averaged to obtain a final value.

### 2.3. Collection and Measurement of Ovarian Samples

At day 30 of the experiment, when the ovarian primordial follicles were largely activated to become growing follicles and small antral follicles, six gilts from each group were randomly selected to collect bilateral ovaries under anesthesia two hours after feeding. On the 19th day of the 3rd estrous cycle, another six gilts from each group were slaughtered by intravenous injection of compound xylazine (0.02 mL/kg BW) 2 hours after feeding. Ovaries were washed with ice-cooled phosphate buffer saline (PBS) three times, dried with heat-sterile tissue paper, and weighed. The number of follicles on the surface of the ovaries were counted on both ovaries. The right ovary was fixed in 4% paraformaldehyde (100 mmol/L phosphate buffer, pH = 7.4). The left ovaries were cut into pieces with scissors, and the ovarian cortex sections were snap-frozen and stored at −80 °C.

### 2.4. Morphological Classification of Follicles

The paraformaldehyde-fixed ovaries were dissected into two parts from the middle with a scalpel, and then embedded in paraffin. Sections were taken at intervals of 50 µm and sections (5 µm) were stained with hematoxylin and eosin following the routine method. The total number of sections analyzed was 20 per ovary (10 sections per paraffin block). Follicle types in ovarian cross-sections were defined and counted as follows. Primordial follicles comprised an oocyte with an obvious nucleus surrounded by a single layer of oblate granulosa cells. Pre-antral follicles comprised an oocyte with an obvious nucleus surrounded by single or multiple layers of cuboidal granulosa cells. Antral follicles were distinguished by the presence of an antrum within the granulosa cell layers enclosing the oocyte and were counted only if the diameter was less than 2 mm in the sections. Follicles were determined to be atretic if they displayed two or more of the following criteria within a single cross-section: more than two pyknotic nuclei, granulosa cells within the antral cavity, granulosa cells pulling away from the basement membrane, or uneven layers of granulosa cells. The area of the ovarian cross-section was measured with Image Pro Plus for Windows (version 6.0; Media Cybernetics, Inc, Maryland, MD, USA). The abundance of each type of follicle was normalized by the volume of ovary tissue in the sections. The images were digitized using a computer coupled to a light microscope with a final magnification of 200× for the primordial and pre-antral follicles, and 100× for the antral. To avoid repeat counting, only those follicles that showed the nucleus of the oocyte were counted.

### 2.5. Detection of Gene Expressions

The total RNA of ovarian granulosa cells and colonic tissues samples was extracted respectively with TRIzol reagent (Invitrogen, Carlsbad, CA, USA). A commercial reverse transcription (RT) kit (TaKaRa, Tokyo, Japan) was used for the synthesis of cDNA. The mRNA levels were then analyzed with a 7900HT Fast Real-Time PCR system (Thermo Fisher Scientific, mTOR, Massachusetts, MA, USA) using synergy brands (SYBR) Green Real-Time PCR regent (RR820A, Takara, Tokyo, Japan). Primers are shown in Table 3. The cycle threshold (2^−ΔΔCt^) method was used to calculate the relative gene expression. β-actin was considered as a house-keeping gene and the outcomes were expressed as fold changes relative to average mRNA levels of genes in the 1.0 DF group.

### 2.6. Western Blot Analysis

For the preparation of protein lysates, about 100 mg ovary tissue powder was homogenized in 1 mL cell lysis buffer (P0013C, Beyotime Biotechnology, Shanghai, China) supplemented with protease inhibitor cocktail (04693132001, Roche, Mannheim, Germany) on a homogenizer. The concentration of protein in the supernatant was measured with a bicinchoninic acid (BCA) Protein Assay Kit (Thermo Fisher Scientific, Illinois, IL, USA). To prepare an electrophoresis sample, 40 µg protein was used with 30 µL loading buffer (1610747, Bio-Rad, California, CA, USA) for each sample. Proteins were separated on 10% polyacrylamide gel, and then transferred onto polyvinylidene fluoride (PVDF) membranes (1620177, Bio-Rad, California, CA, USA). The membranes were blocked in 1% BSA/1× TBST for 1 h at room temperature, followed by incubation with the appropriate primary antibodies (1 µg/mL) overnight. mTOR (2972S), p-mTOR (2971S), S6 (2217S), p-S6 (4858T), AMPK (2793S), p-AMPK (2535S), extracellular regulated protein kinases1/2 (ERK1/2) (9202S), p-ERK1/2 (9201S), Caspase-3 (9662), β-Actin (4970) antibodies were obtained from Cell Signaling Technology (Danvers, MA, USA). After thorough washing, membranes were incubated with appropriate horseradish peroxidase-linked secondary antibodies (7074, Cell Signaling Technology, Beverly, Massachusetts, MA, USA) diluted at 1:2000 in 5% milk in 1× TBST for 1 h. After further thorough washing, protein signals were detected by enhanced chemiluminescence (ECL) western blotting detection reagent (1705060, BioRad, CA, USA) on a Molecular Imager ChemiDoc XRS+ System (Bio-Rad Laboratories). Blots were quantified with ImageJ software (National Institutes of Health, Bethesda, Maryland, MD, USA).

### 2.7. Statistical Analysis

This experiment was designed using a completely randomized design (CRD). The original data was checked by using Grubbs’ test method. If |Xp − $\overset{-}{X}$| > λ (α, n) S, Xp was considered as the outlier. The data were tested to check the homogeneity of variances and normal distribution of the residuals before using parametric analyses. Statistical analyses were performed through Mixed procedure of SAS 9.4 (SAS Institute Inc., Cary, North Carolina, NC, USA) in a completely randomized design. The following statistical model was used: Y_ij_ = μ + T_i_ + e_ij_, where Y is the analyzed variable, μ is the overall mean, T_i_ is the fixed effect of the ith treatment, and e_ij_ is the error term specific to the pig identified assigned to the ith treatment. Orthogonal polynomial contrasts were used to analyze the linear and quadratic effects of the increasing dietary fiber intake levels. Differences were considered statistically significant when *p* < 0.05 and as a trend to significance when 0.05 ≤ *p* < 0.10.

## 3. Results

### 3.1. The Growth Performance and Pubertal Onset

In the present study, all pigs were able to consume the feed provided. The daily intake of digestible energy, amino acids, and the other nutrients were similar between groups with the exception of the linear increase of dietary fiber intake (Table 2). On average, the daily dietary fiber intake was 284.28 g/day, 420.92 g/day, 494.91 g/day, 568.16 g/day for 1.0 DF, 1.5 DF, 1.75 DF, and 2.0 DF groups throughout the experiment period, respectively. The bodyweight at day 30, 60, 90, and 120 of the experiment and the average daily bodyweight gain during different experimental phases were not affected by the consumption of different levels of dietary fiber (*p* > 0.05, Table 4). The age at puberty, bodyweight at puberty, and backfat thickness at puberty were not affected by consumption of different levels of dietary fiber (*p* > 0.05, Table 4).

### 3.2. Development of Ovarian Follicles

The numbers of follicles in different categories of gilts at day 30 of experiment are shown in Table 5. The number of follicles with diameter between 1–3 mm on the surface of the ovaries was increased linearly (*p* < 0.05) and quadratically (*p* = 0.087) as intake of dietary fiber increased.

The percentage of atretic follicles deceased linearly (*p* < 0.05) as intake of dietary fiber increased. There was a tendency for the percentage of primordial follicles (*p* = 0.059) to be affected by dietary fiber levels. If the numbers of follicles were calculated on a volume basis, the number of primordial follicles (*p* < 0.05) and total number of follicles (*p* = 0.059) per cubic centimeter of ovarian tissues increased linearly by dietary fiber levels (Table 5). Notably, the number of atretic follicles decreased linearly with dietary fiber levels (*p* < 0.01, Table 5).

The numbers of follicles in different categories at the 19th day of the 3rd estrous cycle are shown in Table 6. The weight of ovaries had a tendency to decrease with dietary fiber levels (Linearly, *p* = 0.081). The number of follicles with a diameter between 1–3 mm, ≥3 mm, and the number of corpora lutea were not affected by dietary fiber levels (*p* > 0.05). The percentage of primordial follicles had a tendency (*p* = 0.065) to linearly increase with dietary fiber levels. The percentages of antral follicles and atretic follicles linearly decreased with dietary fiber levels (*p* < 0.05). The number of primordial follicles (*p* = 0.054), primary follicles (*p* = 0.010), and total follicles (*p* = 0.073) linearly increased with dietary fiber levels, and the number of atretic follicles linearly decreased with dietary fiber levels (*p* < 0.01, Table 6). The percentages of atretic follicles and antral follicles deceased linearly (*p* < 0.05) as intake of dietary fiber increased. The percentage of primordial follicles had a tendency to increase linearly with dietary fiber levels (*p* = 0.065).

### 3.3. Gene Expressions

**Figure 1:**
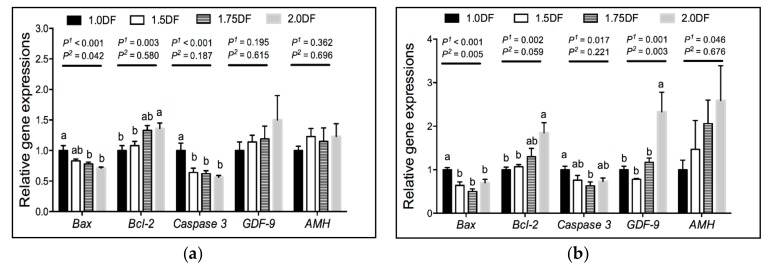
Effects of dietary fiber intake levels on the gene expressions of ovarian tissues in gilts on day 30 of the experiment (**a**) and on the 19th day of the 3rd estrous cycle (**b**). Data are expressed as means ± SE; *n* = 6; DF, dietary fiber; *P*^1^ denotes linear effects; *P*^2^ denotes quadratic effects; ^a,b^ denotes *p* < 0.05.

The expression of genes studied in the ovarian tissues of gilts at day 30 of the experiment and at slaughter (on the 19th day of the 3rd estrous) are presented in Figure 1a,b, respectively. The relative expression of genes *Bax* and *Caspase-*3 were down-regulated linearly (*p* < 0.05) in ovarian tissues of gilts at day 30 of the experiment and at slaughter as the intake of dietary fiber increased (Figure 1a,b). The relative expression of *Bcl-2* in ovarian tissues was up-regulated linearly (*p* < 0.05) in gilts at day 30 of the experiment and at slaughter as the intake of dietary fiber increased (Figure 1a,b). In addition, the relative expression of genes *GDF-9* and *AMH* were up-regulated linearly (*p* < 0.05) in ovarian tissue of gilts at slaughter as the intake of dietary fiber increased (Figure 1b).

### 3.4. Protein Expressions

**Figure 2:**
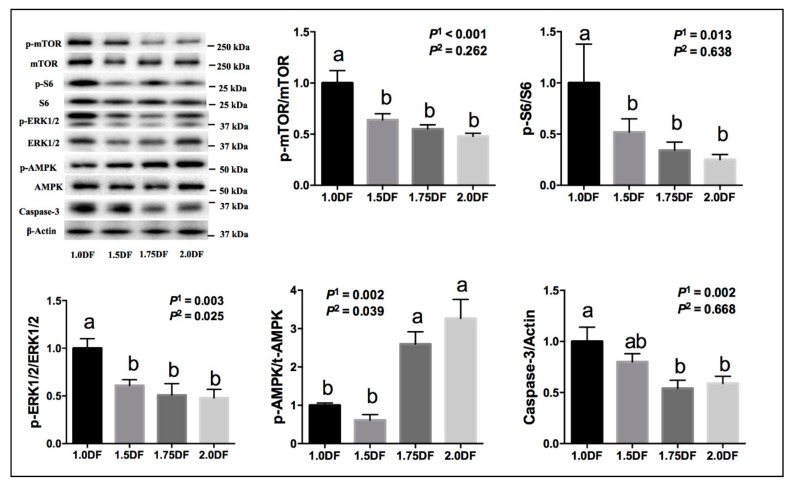
Effects of dietary fiber intake levels on the protein expressions of ovarian tissues in gilts on day 30 of the experiment. Data are expressed as means ± SE; *n* = 6; DF, dietary fiber; *P*^1^ denotes linear effects; *P*^2^ denotes quadratic effects; ^a,b^ denotes *p* < 0.05.

Ovarian protein expressions are shown in Figure 2. The ratios of p-mTOR/mTOR, p-S6/S6, p-ERK_1/2_/ERK_1/2_, and Caspase 3/β-actin in ovarian tissues at day 30 of the experiment were down-regulated linearly or quadratically (*p* < 0.05) as intake of dietary fiber increased. The ratios of p-AMPK/AMPK in ovarian tissues at day 30 of the experiment were up-regulated linearly and quadratically (*p* < 0.05) as the intake of dietary fiber increased.

**Figure 3:**
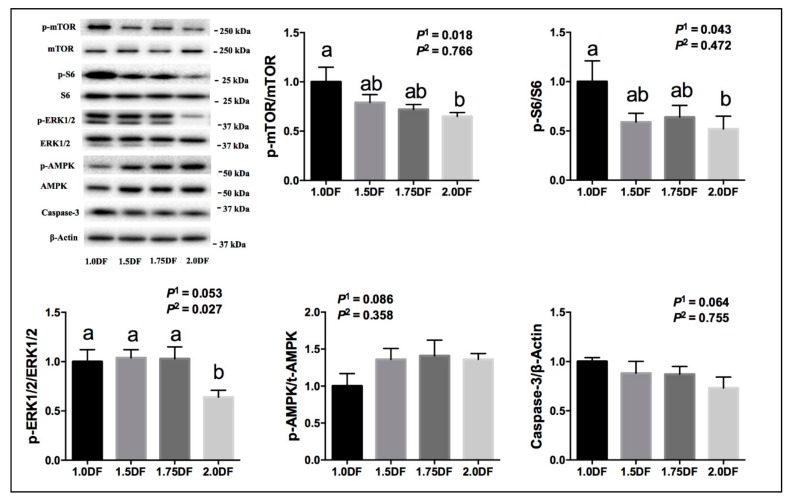
Effects of dietary fiber intake levels on the gene expression of ovarian tissues in gilts on the 19th day of the 3rd estrous cycle. Data are expressed as means ± SE; *n* = 6; DF, dietary fiber; *P*^1^ denotes linear effects; *P*^2^ denotes quadratic effects; ^a,b^ denotes *p* < 0.05.

As shown in Figure 3, the ratios of p-mTOR/mTOR and p-S6/S6 in ovarian tissue at slaughter were down-regulated linearly (*p* < 0.05) as dietary fiber levels increased. The ratios of p-ERK_1/2_/ERK_1/2_ in ovarian tissues at slaughter were up-regulated linearly (*p* = 0.053) and quadratically (*p* < 0.05) as dietary fiber intake levels increased. The ratios of p-AMPK/AMPK had a tendency to be up-regulated linearly (*p* = 0.086), and Caspase 3 had a tendency to be down-regulated linearly (*p* = 0.064) in ovarian tissues at slaughter as intake of dietary fiber increased.

## 4. Discussion

To date, there is no clear optimal level of dietary fiber for replacement gilts in swine production, despite the fact that inclusions of dietary fiber-rich ingredients could enhance the follicle maturation and embryo survival [10,11,13]. In the present study, the effects of dietary fiber levels on the ovarian follicle reserve were investigated in growing replacement gilts and it was found that the activation and survival of follicles were sensitive to the intake of dietary fiber, which shed a light on the importance of dietary fiber for the feeding and management of replacement gilts.

It is clear that the digestibility of nutrients such as energy and amino acids, might be decreased by dietary fiber. Nevertheless, the intake of nutrients, except dietary fiber, was not affected by the addition of dietary fiber and the growth performance of gilts was not affected dietary fiber levels. This could be the reason why the pubertal onset was not affected dietary fiber levels, since pubertal onset was largely dependent on bodyweight and body composition of gilts [15,16]. However, in our previous research, a soluble fiber supplementation was observed to enhance the pubertal onset and the subsequent reproductive performance without the changes of bodyweight [12]. The difference of the results between the two studies might be explained by the difference in the type of dietary fiber.

Although the pubertal onset was not altered by the dietary fiber levels, the ovarian follicular development was significantly changed by dietary treatment. The ovarian reserve, the storage of the female germline, plays a critical role in determining the reproductive span for both humans and rodent animals [17]. In the present study, the percentage of ovarian primordial follicles, the most important fraction of the female ovarian reserve, ranged between 63% to 71% and 56% to 68% among groups at day 30 of the experiment and the 19th day of the 3rd estrous cycle, respectively. Our results agreed with previous findings, which found that primordial follicles accounted for 63.39% of total follicles in young adult gilts [18]. Thus, the primordial follicles could represent the majority of the female germline for young adult gilts due to their higher percentage in the ovary. In the present study, the reserve of the primordial follicles was significantly elevated by dietary fiber, which might be ascribed to the lower activation ratio of those follicles. Within each ovary, the ovarian primordial follicles remained quiescent, unless being activated to become primary follicles [19]. The fate of primordial follicles, quiescence or activation, was largely dependent on the cellular nutrient sensors mTOR/S6, which served as the central determinant of activation of primordial follicles [5,20]. Recent findings in our lab revealed that mTORC1 signaling was closely associated with changes of dietary macronutrient balance, and mTORC1 signaling mediated the effects of nutrient balance on the activation or survival of primordial follicles [6]. Our current results have demonstrated that the ovarian phosphorylation of mTOR and its downstream target S6 linearly decreased with dietary fiber. Correspondingly, the ERK1/2 signaling, which was closely associated with mTORC1 signaling and involved in primordial follicle activation [21], was also inhibited with the increase of dietary fiber levels, suggesting that intake of increased dietary fiber levels was able to retain a larger primordial follicle reserve. Results in both humans and rodent animals revealed that the quiescence of primordial follicles must be strictly regulated to prevent premature exhaustion of the primordial follicle reserve, which results in termination of the reproductive processes and reproductive aging [5,6]. Following activation, primordial follicles transit through growth stages and subsequently provide the mature oocytes through the processes of folliculogenesis. Interestingly, although the activation of primordial follicles was decreased by dietary fiber, the folliculogenesis was not negatively affected because a greater number of antral follicles with a diameter between 1–3 mm at day 30 of the experiment was observed for gilts with high dietary fiber intake. Such findings were consistent with the results obtained in cattle which found that the number of primordial follicles predicted a greater number of large antral follicles [22]. However, the number of large antral follicles at the 19th day of the 3rd estrous cycle was not affected by dietary fiber levels. Previous results found that the correlation between the primordial follicle reserve and number of antral follicles was different between species, and the number of primordial follicles could only be positively corrected with antral follicle counts in cows, but not in porcine [18]. Nevertheless, our results demonstrated that the activation of primordial follicles was decreased by dietary fiber, without the negative effects on folliculogenesis for gilts.

Additionally, one of the most important finding in the present study was that extra consumption of dietary fiber resulted in substantial reductions of atretic follicles. Follicle atresia mostly occurred in the secondary follicles and early antral follicles, which was initiated by a series of apoptotic factors [23,24]. The expression of pro-apoptotic factor genes *Bax* and *Caspase* 3 were linearly down-regulated by dietary fiber levels, while mRNA expression of *Bcl-*2, which encoded a BCL-2 protein endowed with anti-apoptotic properties, was linearly up-regulated by dietary fiber levels, indicating that the nutritional signals of dietary fiber exerted significant anti-apoptotic effects on ovarian follicles. To date, there is a paucity of data elucidating the relationship between dietary fiber and the ovarian follicle reserve, as well as the underlying mechanisms, in pigs. A previous review summarized that the cellular activation of AMPK by the dietary fiber derived short-chain fatty acids was the main mechanism mediating the beneficial effects of dietary fiber on host metabolism and physiology [25]. A close relationships between AMPK and mTOR signaling and Caspase 3 signaling had been observed [26,27]. In the present study, we did observe a linear increases of AMPK phosphorylation in ovarian tissues in response to dietary fiber levels, which might be the reason why ovarian mTORC1 signaling and Caspase-3 expression were changed by dietary fiber. However, concentrations of short-chain fatty acids in ovarian tissues (e.g., follicular fluid) were quite low in our attempts to detect their existence, therefore, the exact pathway(s) mediating the beneficial effects of dietary fiber on the follicle reserve needs further investigation.

## 5. Conclusions

In conclusion, consumption of a greater amount of dietary fiber could result in a larger ovarian follicle reserve, which was associated with a series of cellular components involving mTORC1, AMPK, and Caspase 3 signaling. Current work revealed that the average daily intake of 494.9 g dietary fiber during from age of 92 days to the 19th day of the 3rd estrous cycle was able to retain a greater number of ovarian follicles reserved for replacement gilts.

## Figures and Tables

**Table 1:** Ingredients and chemical composition of basal diets (g/kg, as fed basis).

Ingredients (g/kg)	1–60 Days				61 Days–Slaughter ^4^			
	1.0 DF ^5^	1.5 DF	1.75 DF	2.0 DF	1.0 DF	1.5 DF	1.75 DF	2.0 DF
Corn	720	720	720	720	780	780	780	780
Soybean (44%CP)	208	208	208	208	160	160	160	160
Fish meal (65%CP)	25	25	25	25	20	20	20	20
Soybean oil	20	20	20	20	17	17	17	17
L-Lys HCl (98%)	3	3	3	3	2	2	2	2
DL-Methionine (99%)	1	1	1	1	0.4	0.4	0.4	0.4
L-Threonine (98%)	0.6	0.6	0.6	0.6	0.2	0.2	0.2	0.2
L-Trptophan (98%)	0.1	0.1	0.1	0.1	0	0	0	0
Choline chloride (50%)	1.5	1.5	1.5	1.5	1.5	1.5	1.5	1.5
Salt	4	4	4	4	4	4	4	4
Limestone	6.2	6.2	6.2	6.2	5.9	5.9	5.9	5.9
Monocalcium phosphate	8.6	8.6	8.6	8.6	7	7	7	7
Vitamin-mineral premix ^1^	2	2	2	2	2	2	2	2
Dietary fiber mixture ^2^	0	63	94	125	0	62	93	124
Total	1000	1063	1094	1125	1000	1062	1093	1124
Nutrient composition, g/kg ^3^								
Digestible energy, Mcal/kg	3.40	3.20	3.11	3.02	3.40	3.20	3.11	3.02
Crude protein	169.0	159.0	154.5	150.2	147.0	138.4	134.5	130.8
Total Lysine	10.8	10.2	9.9	9.6	8.6	8.1	7.9	7.6
Standardized ileal digestible lysine	9.8	9.2	9.0	8.7	7.8	7.3	7.1	6.9
Calcium	6.9	6.5	6.3	6.1	5.9	5.6	5.4	5.2
Total phosphorus	5.9	5.6	5.4	5.2	5.3	5.0	4.8	4.7
Soluble fiber	10.2	21.4	26.5	31.3	10.3	21.4	26.4	31.2
Insoluble fiber	115.0	155.6	173.8	191.1	113.9	154.0	172.3	189.6
Total dietary fiber ^3^	125.2	177.0	200.4	222.4	124.2	175.3	198.7	220.8

^1^ Provided the following per kilogram of basal diet: 8000 IU vitamin A, 800 IU vitamin D_3_, 30 IU vitamin E, 4 mg vitamin K, 0.16 mg biotin, 2 mg folacin, 25 mg niacin, 20 mg pantothenic acid, 10 mg riboflavin, 2 mg thiamin, 1 mg vitamin B6, 20 µg vitamin B12, 16 mg copper, 0.25 mg iodine, 125 mg iron, 30 mg manganese, 0.25 mg selenium, 125 mg zinc. ^2^ Dietary fiber mixture was composed of inulin and cellulose, with a ratio of 1:4. ^3^ Total dietary fiber = soluble fiber + insoluble fiber, analyzed value according to method AOAC 991.43. ^4^ Gilts were slaughtered on the 19th day of the 3rd estrous cycle. ^5^ DF, dietary fiber.

**Table 2:** The daily nutrient intake of the gilts during different feeding phases.

Nutrient Intake	1.0 DF ^1^	1.5 DF	1.75 DF	2.0 DF
Day 1 to 30 of experiment (g/day)				
Feed intake	1600	1700	1750	1800
DE ^2^ intake (Mcal/day)	5.44	5.44	5.44	5.44
Total lysine intake	17.28	17.28	17.28	17.28
Soluble fiber intake	16.32	36.38	46.38	56.34
Insoluble fiber intake	184.00	264.52	304.15	343.98
Total dietary fiber (soluble + insoluble) intake	200.32	300.90	350.7	400.32
Day 31 to 60 of experiment (g/day)				
Feed intake	2100	2232	2297	2362
DE intake (Mcal/day)	7.14	7.14	7.14	7.14
Total lysine intake	22.68	22.68	22.68	22.68
Soluble fiber intake	21.42	47.76	60.87	73.93
Insoluble fiber intake	241.50	347.30	399.22	451.38
Total dietary fiber (soluble + insoluble) intake	262.92	395.06	460.32	525.31
Day 61 to 120 of experiment (g/day)				
Feed intake	2500	2655	2732	2810
DE intake (Mcal/day)	8.5	8.5	8.5	8.5
Total lysine intake	21.5	21.5	21.5	21.5
Soluble fiber intake	25.75	56.82	72.12	87.67
Insoluble fiber intake	284.75	408.87	470.72	532.78
Total dietary fiber (soluble + insoluble) intake	310.5	465.42	542.85	620.45
Day 121 to slaughter ^3^ (g/day)				
Feed intake	2800	2974	3060	3174
DE intake (Mcal/day)	9.52	9.52	9.52	9.52
Total lysine intake	24.08	24.08	24.08	24.08
Soluble fiber intake	28.84	63.64	80.78	99.03
Insoluble fiber intake	318.92	458.00	527.24	601.79
Total dietary fiber (soluble + insoluble) intake	347.76	521.34	608.02	700.82

^1^ DF, dietary fiber. ^2^ DE, digestible energy. ^3^ Gilts were slaughtered at the 19th day of the 3rd estrous cycle.

**Table 3:** Oligonucleotide primers used for real-time quantitative PCR analysis.

Primers	Sequences (5′–3′)	Gene Bank No.
Bax	F: CGCATTGGAGATGAACTGGA R: CCAGTTGAAGTTGCCGTCAG	XM_003127290.5
Bcl-2	F: GCCTTTGTGGAGCTGTATGG R: CCCGTGGACTTCACTTATGG	XM_021099593.1
Caspase-3	F: GCCGAGGCACAGAATTGGACTG R: GCCAGGAATAGTAACCAGGTGCTG	NM_214131.1
GDF-9	F: GGTATGGCTCTCCGGTTCACAC R: CTTGGCAGGTACGCAGGATGG	NM_001001909.1
AMH	F: GACTCTGGCTTCCTGGCGTTG R: ATCCGTGTGAAGCAGCGAGAG	NM_214310.3
β-actin	F: CCAGCACGATGAAGATCAAGA R: AATGCAACTAACAGTCCGCCTA	XM_003124280.5

Bax, bcl2-associated X protein; Bcl-2, B-cell lymphoma-2; GDF9, Growth differentiation factor 9; AMH, anti-Müllerian hormone.

**Table 4:** Effects of dietary fiber levels on growth performance and pubertal onset in gilts.

Items	Treatments				*p*-Value	
	1.0 DF	1.5 DF	1.75 DF	2.0 DF	Linear	Quadratic
Initial age, days	92.7 ± 0.2	92.6 ± 0.1	92.6 ± 0.2	92.6 ± 0.1	0.810	0.709
BW, kg						
Initial	33.8 ± 0.8	33.8 ± 0.9	33.8 ± 0.9	33.8 ± 0.9	0.980	0.994
Day 30	54.1 ± 0.9	53.6 ± 0.7	53.6 ± 0.8	53.1 ± 0.8	0.397	0.938
Day 60	75.6 ± 1.3	76.2 ± 1.0	75.9 ± 1.2	76.0 ± 1.2	0.826	0.814
Day 90	99.3 ± 1.3	100.0 ± 1.4	99.8 ± 1.2	100.3 ± 1.4	0.634	0.999
Day 120	120.9 ± 1.7	121.5 ± 1.6	121.2 ± 1.5	121.4 ± 1.5	0.861	0.892
ADG, kg/day						
Day 0–30	0.68 ± 0.02	0.66 ± 0.02	0.66 ± 0.01	0.64 ± 0.02	0.173	0.893
Day 30–60	0.72 ± 0.03	0.74 ± 0.03	0.73 ± 0.04	0.75 ± 0.02	0.613	0.964
Day 60–90	0.79 ± 0.04	0.79 ± 0.02	0.80 ± 0.02	0.81 ± 0.03	0.651	0.735
Day 90–120	0.74 ± 0.03	0.72 ± 0.03	0.74 ± 0.02	0.72 ± 0.02	0.721	0.720
ADFI, kg/day	2.41 + 0.01 ^d^	2.56 + 0.01 ^c^	2.65 + 0.01 ^b^	2.73 + 0.01 ^a^	<0.001	0.837
BF at day 30, mm	9.63 ± 0.18	9.55 ± 0.20	9.50 ± 0.22	9.53 ± 0.22	0.669	0.870
BF at day 120, mm	14.56 ± 0.43	14.41 ± 0.38	13.91 ± 0.44	14.15 ± 0.35	0.332	0.848
Age at puberty, days	198.3 ± 6.4	190.4 ± 4.1	195.5 ± 4.0	197.3 ± 7.0	0.627	0.612
BW at puberty, kg	110.6 ± 4.2	108.0 ± 4.4	111.7 ± 3.7	113.1 ± 5.7	0.682	0.552
BF at puberty, mm	13.32 ± 0.47	12.67 ± 0.51	12.84 ± 0.46	13.19 ± 0.44	0.773	0.289

N = 19 per group before day 30 of experiment, and *n* = 13 after day 30 of experiment. Data are expressed as means ± SE; DF, dietary fiber; BW, bodyweight; ADG, average daily bodyweight gain; ADFI, average daily feed intake; BF, backfat thickness; ^a,b,c,d^ Means within a row with different superscripts differ (*p* < 0.05).

**Table 5:** Effects of dietary fiber levels on development of follicles in gilts at day 30 of the experiment.

Items	Treatments				*p*-Value	
	1.0 DF	1.5 DF	1.75 DF	2.0 DF	Linear	Quadratic
Weight of ovaries, g	7.85 ± 0.57	7.46 ± 1.02	6.35 ± 0.45	6.63 ± 0.66	0.154	0.951
Relative weight of ovaries, g/kg	0.114 ± 0.09	0.116 ± 0.013	0.094 ± 0.005	0.103 ± 0.011	0.266	0.929
No. of follicle with diameter of 1–3 mm, n	125.8 ± 5.8 ^b^	131.7 ± 11.3 ^ab^	130.4 ± 12.3 ^ab^	166.5 ± 9.7 ^a^	0.020	0.087
Follicle percentage						
Primordial follicle	63.65 ± 2.14	64.72 ± 3.19	66.12 ± 3.26	71.67 ± 1.52	0.059	0.264
Primary follicle	11.41 ± 0.79	9.62 ± 0.47	10.57 ± 1.34	9.51 ± 0.70	0.196	0.699
Secondary follicle	9.17 ± 0.45	10.03 ± 1.67	9.78 ± 1.16	8.66 ± 0.82	0.848	0.364
Antral follicle	8.85 ± 2.30	9.76 ± 1.26	9.24 ± 1.80	7.36 ± 0.51	0.596	0.347
Atretic follicle	6.91 ± 0.76 ^a^	5.86 ± 0.91 ^a^	4.28 ± 0.62 ^ab^	2.82 ± 0.42 ^b^	<0.001	0.278
Number of ovarian follicles, 10^3^/cm						
Primordial follicle	40.17 ± 2.61 ^b^	43.34 ± 6.45 ^b^	45.77 ± 3.28 ^ab^	64.51 ± 10.59 ^a^	0.028	0.147
Primary follicle	7.36 ± 1.03	6.23 ± 0.64	7.32 ± 0.98	8.45 ± 1.24	0.474	0.190
Secondary follicle	5.84 ± 0.61	6.26 ± 0.76	6.90 ± 1.00	7.61 ± 1.27	0.190	0.688
Antral follicle	5.79 ± 1.63	6.04 ± 0.63	6.47 ± 1.41	6.35 ± 0.71	0.688	0.956
Atretic follicle	4.47 ± 0.65 ^a^	3.67 ± 0.40 ^ab^	2.96 ± 0.41 ^bc^	2.33 ± 0.22 ^c^	0.002	0.662
Total follicles	63.64 ± 5.08 ^b^	65.55 ± 7.03 ^ab^	69.42 ± 3.91 ^ab^	89.25 ± 13.43 ^a^	0.059	0.183

^a,b,c^ Means within a row with different superscripts differ (*p* < 0.05).

**Table 6:** Effects of dietary fiber levels on development of follicles in gilts on the 19th day of the 3rd estrous cycle.

Items	Treatments				*p*-Value	
	1.0 DF	1.5 DF	1.75 DF	2.0 DF	Linear	Quadratic
BW at slaughter, kg	146.7 ± 1.6	145.3 ± 1.8	145.8 ± 3.0	142.3 ± 1.7	0.209	0.549
Weight of ovaries, g	16.78 ± 0.71	14.96 ± 0.62	14.58 ± 0.75	14.88 ± 1.15	0.081	0.341
Relative weight of ovaries, g/kg	0.114 ± 0.004	0.103 ± 0.004	0.100 ± 0.005	0.105 ± 0.009	0.186	0.287
No. of follicle, n						
Diameter 1–3 mm	25.8 ± 3.4	25.0 ± 5.4	33.8 ± 6.5	31.7 ± 10.1	0.424	0.856
Diameter ≥3 mm	39.5 ± 3.5	40.0 ± 1.3	37.3 ± 5.7	40.7 ± 1.9	0.978	0.773
No. of corpora lutea, n	21.2 ± 1.8	24.7 ± 1.7	29.5 ± 2.5	22.8 ± 3.0	0.243	0.102
Follicle percentage, %						
Primordial follicle	56.93 ± 5.18	63.37 ± 2.00	63.07 ± 3.17	68.12 ± 4.32	0.065	0.961
Primary follicle	12.05 ± 1.15	10.93 ± 1.11	12.73 ± 1.22	11.67 ± 2.22	0.976	0.839
Secondary follicle	9.01 ± 2.08	10.99 ± 0.84	9.11 ± 0.34	9.07 ± 1.27	0.934	0.317
Antral follicle	15.25 ± 2.31	10.39 ± 1.57	11.06 ± 1.27	9.48 ± 0.82	0.020	0.449
Atretic follicle	6.75 ± 1.00 ^a^	4.40 ± 0.41 ^ab^	3.91 ± 0.92 ^a,b^	2.42 ± 0.46 ^b^	0.001	0.989
Number of ovarian follicles, 10^3^/cm						
Primordial follicle	9.88 ± 1.40 ^b^	16.20 ± 1.78 ^ab^	19.33 ± 3.60 ^a^	18.55 ± 4.20 ^ab^	0.054	0.494
Primary follicle	2.72 ± 0.38 ^bc^	2.52 ± 0.20 ^c^	3.50 ± 0.29 ^ab^	3.84 ± 0.27 ^a^	0.010	0.110
Secondary follicle	2.21 ± 0.69	2.65 ± 0.26	2.72 ± 0.28	3.05 ± 0.47	0.210	0.957
Antral follicle	3.37 ± 0.56	2.58 ± 0.40	3.11 ± 0.37	3.18 ± 0.54	0.818	0.318
Atretic follicle	1.48 ± 0.20 ^a^	1.09 ± 0.12 ^ab^	1.05 ± 0.15b	0.74 ± 0.05 ^b^	0.002	0.839
Total follicles	18.97 ± 3.06	25.04 ± 2.06	29.71 ± 4.21	28.34 ± 4.71	0.073	0.593

Data are expressed as means ± SE; DF, dietary fiber; n = 6; ^a,b,c^ Means within a row with different superscripts differ (*p* < 0.05).
